# Th17 and Th22 cells in psoriatic arthritis and psoriasis

**DOI:** 10.1186/ar4317

**Published:** 2013-09-26

**Authors:** Helen Benham, Paul Norris, Jane Goodall, Mihir D Wechalekar, Oliver FitzGerald, Agnes Szentpetery, Malcolm Smith, Ranjeny Thomas, Hill Gaston

**Affiliations:** 1 The University of Queensland Diamantina Institute, Translational Research Institute, 37 Kent Street, Woolloongabba QLD 4102, Australia; 2 Department of Dermatology Addenbrooke’s Hospital, Hills Road, Cambridge CB2 0QQ, UK; 3 Department of Medicine, University of Cambridge, Addenbrooke’s Hospital, Hills Road, Cambridge CB2 0QQ, UK; 4 Rheumatology Unit, Repatriation General Hospital, 216 Daws Rd, Daw Park, South Australia 5042, Australia; 5 Flinders University, Bedford Park, Sturt Rd, South Australia 5042, Australia; 6 Department of Rheumatology, St Vincent’s University Hospital, Merrion Rd, Dublin 4, and The Conway Institute for Biomolecular Research, University College Dublin, Belfield, Dublin 4, Dublin, Ireland

## Abstract

**Introduction:**

The aim of this study was to characterize interleukin 17 (IL-17) and interleukin 22 (IL-22) producing cells in peripheral blood (PB), skin, synovial fluid (SF) and synovial tissue (ST) in patients with psoriasis (Ps) and psoriatic arthritis (PsA).

**Methods:**

Flow cytometry was used to enumerate cells making IL-22 and IL-17, in skin and/or SF and PB from 11 patients with Ps and 12 patients with PsA; skin and PB of 15 healthy controls and SF from rheumatoid arthritis (RA) patients were used as controls. Expression of the interleukin 23 receptor (IL-23R) and chemokine receptors CCR4 and CCR6 was examined. Secretion of IL-17 and IL-22 was measured by ELISA. ST was analysed by immunohistochemical staining of IL-17 and IL-22.

**Results:**

Increased frequencies of IL-17+ and IL-22+ CD4+ T cells were seen in PB of patients with PsA and Ps. IL-17 secretion was significantly elevated in both PsA and Ps, whilst IL-22 secretion was higher in PsA compared to Ps and healthy controls. A higher proportion of the CD4+ cells making IL-17 or IL-22 expressed IL-23R and frequencies of IL-17+, CCR6+ and CCR4+ T cells were elevated in patients with Ps and those with PsA. In patients with PsA, CCR6+ and IL-23R + T cells numbers were elevated in SF compared to PB. Increased frequencies of IL-17+ and IL-22+ CD4+ T cells were demonstrated in Ps skin lesions. In contrast, whilst elevated frequencies of CD4+ IL-17+ cells were seen in PsA SF compared to PB, frequencies of CD4+ IL-22+ T cells were lower. Whereas IL-17 expression was equivalent in PsA, osteoarthritis (OA) and RA ST, IL-22 expression was higher in RA than either OA or PsA ST, in which IL-22 was strikingly absent.

**Conclusions:**

Elevated frequencies of IL-17 and IL-22 producing CD4+ T cells were a feature of both Ps and PsA. However their differing distribution at disease sites, including lower frequencies of IL-22+ CD4+ T cells in SF compared to skin and PB, and lack of IL-22 expression in ST suggests that Th17 and Th22 cells have common, as well as divergent roles in the pathogenesis of Ps and PsA.

## Introduction

Psoriasis (Ps) is a common inflammatory disease of the skin affecting 1% to 3% of the population [1-3]. It is complicated in up to 30% of cases by psoriatic arthritis (PsA) [4]. The arthritis takes various forms and is a member of the spondyloarthropathies (SpAs) [5]. Ps alone produces significant disability; when combined with PsA, the condition can be especially debilitating, and treatment for both skin and joints remains suboptimal.

Whereas recent evidence implicates interleukin 22 (IL-22) in the pathogenesis of skin disease in Ps [6,7], PsA has been postulated to more likely involve IL-17 [8,9]. Both cytokines can be made by the T helper 17 (Th17) cell subset, but recent reports have described T cells that make IL-22 alone [10,11]. These T cells, subsequently termed *Th22 cells*, produce IL-22 without IL-17 or interferon γ (IFNγ) and are characterized by the expression of certain chemokine receptors, including chemokine receptor 4 (CCR4) and CCR6, which can influence homing to skin and joints [12]. Both Th17 and Th22 cells are influenced by the cytokine IL-23, which is required for their expansion and maintenance [13]. Genetic studies implicate IL-23 in Ps and PsA [14].

Despite increasing evidence of their involvement in Ps and PsA, the relative roles of Th22 and Th17 cells in these conditions are not known. In this study, we characterized cells making IL-22 and/or IL-17 in skin, synovial fluid (SF), synovial tissue (ST) and peripheral blood (PB) of Ps and PsA patients, together with PB and skin from healthy controls and SF from rheumatoid arthritis (RA) patients. We examined the expression of IL-23 receptor (IL-23R) and the chemokine receptors CCR4 and CCR6, which influence traffic of these cells into skin and joints. ST expression of IL-17 and IL-22 was analysed by immunohistochemical staining.

## Methods

### Patients

PB samples were obtained from 12 patients with Ps, 11 patients with PsA and 15 healthy controls. Skin biopsies (4 mm) were obtained from seven patients with Ps and healthy skin samples from seven patients undergoing plastic surgical procedures. SF samples were collected from seven patients with PsA and six patients with RA. All patients with PsA fulfilled the Classification Criteria for Psoriatic Arthritis criteria [15], and Ps patients were diagnosed by a consultant dermatologist. Demographics and disease parameters were recorded (Table 1). The study was approved by the Addenbrooke’s Hospital and Repatriation General Hospital local ethics committee, and written informed consent was given by all patients.

**Table 1:** **Baseline clinical and demographic characteristics of psoriatic arthritis patients, psoriasis patients and healthy donors**^**a**^

**Patient characteristics**	**Psoriatic arthritis (*N *** **= 11)**	**Psoriasis (*N *** **= 12)**	**Healthy donors (*N *** **= 15)**
Age, mean ± SD (years)	52 ± 17	51 ± 19	43 ± 12
Sex, F/M	6/5	4/8	9/6
Duration of arthritis, mean ± SD (years)	11 ± 2	NA	NA
Duration of psoriasis, mean ± SD (years)	18 ± 8	25 ± 12	NA
Current treatment	Nil = 2	Nil = 1	Nil
	Steroids = 1	Topical steroids = 4	
	Methotrexate = 4	PUVA = 4	
	Sulphasalazine = 2	Methotrexate = 1	
	Azathioprine = 1	Acitretin = 2	
	Etanercept = 1	Cyclosporine = 1	
	Adalimumab = 1		
	Infliximab = 1		
PASI score, mean ± SD	12 ± 6	13 ± 7	**0**
Joint count, mean ± SD	5 ± 13	NA	**0**

^a^NA, Not applicable; PASI, Psoriasis Area and Severity Index; PUVA, Psoralen and ultraviolet A light phototherapy.

### Preparation and stimulation of PBMCs and SFMCs

Peripheral blood mononuclear cells (PBMCs) and synovial fluid mononuclear cells (SFMCs) were purified from PB and SF by centrifugation using a Ficoll-Hypaque gradient (GE Healthcare Biosciences AB, Uppsala, Sweden). PBMCs and SFMCs were adjusted to a final concentration of 10^6^/ml in RPMI 1640 medium with 10% heat-inactivated foetal calf serum, 1% glutamine/penicillin/streptomycin and 2% 2-[4-(2-hydroxyethyl)piperazin-1-yl]ethanesulphonic acid (HEPES). For surface phenotype and intracellular cytokine staining, PBMCs and SFMCs were seeded into 24-well plates (Nalge Nunc, Roskilde, Denmark) at 2 × 10^6^ cells/well and stimulated *ex vivo* with phorbol 12-myristate 13-acetate (50 ng/ml; Calbiochem, Nottingham, UK) and calcium ionomycin (1 μg/ml; Sigma-Aldrich, St Louis, MO, USA) for five hours. GolgiStop protein transport inhibitor (BD Biosciences, Mountain View, CA, USA) was added at the beginning of the stimulation.

### Cytokine secretion

PBMCs were seeded into 96-well culture plates (Nalge Nunc) at 10^5^/200 μl/well in triplicate and stimulated with anti-CD3/CD28 beads (10^5^ beads/well; Invitrogen, Oslo, Norway). Following incubation for four days, cell-free supernatants were collected and the concentrations of IL-17 and IL-22 were assessed using enzyme-linked immunosorbent assay kits according to the manufacturer’s instructions (eBioscience, San Diego, CA, USA). The detection limits were 4 pg/ml for IL-17 and 8 pg/ml for IL-22.

### Dermal single-cell suspensions

Dermal single-cell suspensions were obtained from skin samples following overnight incubation in dispase and collagenase 1 mg/ml at 4°C (both from Invitrogen, Paisley, UK). Epidermis and dermis samples were separated, and the dermis was cultured for 36 to 48 hours at 37°C in RPMI 1640 medium supplemented with 5% pooled human serum (First Link, Birmingham, UK), 0.1% gentamicin reagent solution (Gibco, Grand Island, NY, USA) and 1% 1 mol/L HEPES buffer (Sigma-Aldrich, Irvine, UK). Dermal single-cell suspensions were stimulated as described for PBMCs and SFMCs.

### Flow cytometry

Flow cytometry was used to analyse surface phenotype and intracellular cytokine production by PBMCs, SFMCs and skin-derived mononuclear cells. Cells were stained with antibodies against surface antigens and intracellular cytokines as previously described [16]. Live CD4+ T cells were gated, and the percentages of these cells producing IL-17, IFNγ and IL-22 were calculated. Skin cells were stained with LIVE/DEAD® Fixable Near-IR Dead Cell Stain Kit (Invitrogen, Oregon, USA) to exclude dead cells from analysis. The FACSCanto II Flow Cytometry System (BD Biosciences) and FlowJo software (Tree Star, Ashland, OR, USA) were used for analysis. Antibodies used were allophycocyanin-cyanine 7 (Cy7)-labelled anti-CD3 (BioLegend, San Diego, CA, USA), phycoerythrin (PE)-Cy7-labelled anti-CD4, PE-Cy5-labelled αβ T-cell receptor (eBioscience), biotin-labelled anti-IL-23R (R&D Systems, Minneapolis, MN, USA) used with Qdot 605 streptavidin conjugate (Invitrogen), PE-labelled anti-CCR6 (BD Biosciences), peridinin-chlorophyll/Cy5.5-labelled anti-CCR4 (BioLegend), fluorescein isothiocyanate-labelled anti-IL-17, eFluor 450-labelled anti-IFNγ (eBioscience) and Alexa Fluor 647-labelled anti-IL-22 (Molecular Probes, Eugene, OR, USA). Appropriately conjugated immunoglobulin G (IgG) antibodies were used as isotype controls.

### Synovial tissue

ST samples from RA, PsA and OA patients were obtained at the time of knee arthroscopy or total knee replacement surgery at the Rheumatology Unit of the Repatriation General Hospital, Daw Park, South Australia, Australia. ST samples were snap-frozen in Tissue-Tek OCT compound (Miles Laboratories, Elkhart, IN, USA) and stored at −80°C. Cryostat sections (6 μm) were cut and mounted on adhesive glass slides (Knittelglaser, Braunschweig, Germany).

### Immunohistochemistry

Serial sections were stained with the following primary antibodies: rabbit polyclonal anti-human IL-17Ab (Santa Cruz Biotechnology, Santa Cruz, CA, USA), rabbit polyclonal anti-human IL-22Ab (Abcam, Cambridge, UK) and rabbit polyclonal anti-human IL-23Ab (Abcam). We used 3 μg/ml goat anti-rabbit IgG (P0448; Dako, Glostrup, Denmark) as a secondary antibody and 7 μg/ml swine anti-goat IgG (ACI3404; Invitrogen) or 13 μg/ml rabbit anti-swine IgG (P0164; Dako) as tertiary antibody. We used a previously described [17-19] three-step peroxidase-based immunohistochemical staining technique for IL-22 and IL-23. For staining of IL-17, biotinylated tyramine was used for amplification with the tyramine signal amplification TSA Biotin System kit (PerkinElmer, Waltham, MA, USA) as previously described [20]. Controls were included in each histochemical labelling run; negative control irrelevant/isotype-matched immunoglobulins were applied to the sections instead of the primary antibody, or the primary antibody was omitted.

### Semiquantitative scoring analysis of immunohistochemistry results

After the slides were stained, the tissue sections were measured by two independent observers using semiquantitative analysis. IL-17 and IL-22 staining was scored using a five-point scale (0 to 4) scoring system as previously described [21]. Assessment was carried out according to the percentage of positively stained cells as follows: 0 = no staining, 1 = <10%, 2 = 11% to 25%, 3 = 26% to 50% and 4 = >50%.

### Statistical analysis

All data are presented as the mean ± SEM. An unpaired Student’s *t*-test was used to detect differences between the means of two normally distributed groups, and the Mann–Whitney *U* test was applied for non–normally distributed groups. One-way or two-way analysis of variance with the Bonferroni multiple comparison *post hoc* test was used to compare multiple means. Pearson’s correlation coefficient was used to test the correlations. Significance values shown on the figures are as follows: *P < 0.05, **P < 0.01 and ***P < 0.001. All analyses were performed using GraphPad Prism 5 software (GraphPad Software, La Jolla, CA, USA).

## Results

### Frequency of IL-17+ and IL-22+CD4+ T cells are increased in PBMCs of patients with both PsA and Ps

**Figure 1:**
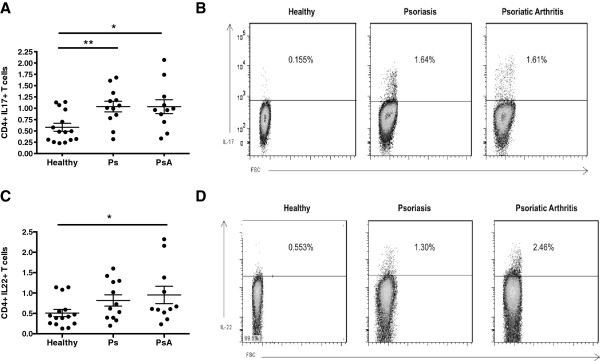
**Frequency of interleukin-17+ and interleukin-22+CD4+ T cells in the peripheral blood mononuclear cells of patients with psoriatic arthritis patients with psoriasis and healthy controls.** The frequency of interleukin 17 (IL-17)-positive CD4+ T cells **(A)** and IL-22-positive CD4+ T cells **(C)** in patients with psoriasis (Ps) or psoriatic arthritis (PsA) and healthy controls. The percentages of total CD4+ T cells are shown. Typical examples of intracellular staining of IL-17 **(B)** and IL-22 **(D)** are also shown after gating for CD3+CD4+ T cells. * p<0.05, **p<0.01, ***p<0.001 FSC = Forward Scatter.

Using flow cytometry, we evaluated intracellular expression of IL-17 and IL-22 and observed a higher proportion of IL-17-producing cells within the PBMCs of PsA and Ps patients compared with that of healthy controls (1.0% and 1.1% vs. 0.57%, respectively; *P* < 0.05, *P* < 0.01, respectively) (Figure 1A). The percentages of IL-22-positive CD4+ T cells were also increased in PBMCs from patients with PsA compared with those of healthy controls (0.95% vs. 0.51%; *P* < 0.05), but the percentage increase in Ps patients relative to controls (0.81% vs. 0.51%; *P* > 0.05) was not statistically significant (Figure 1C). There were no differences in the frequency of IL-17+ and IL-22+CD4+ T cells between patients with Ps alone and those with PsA (Figures 1A and 1C).

### Increased secretion of IL-17 and IL-22 by PBMCs from psoriatic arthritis and psoriasis patients

**Figure 2:**
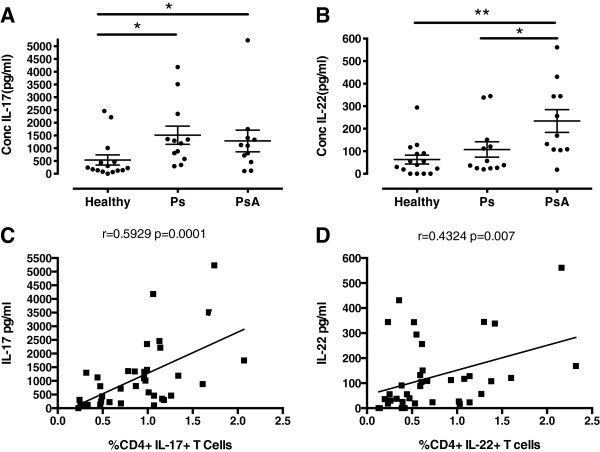
**Secretion of interleukin 17 and interleukin 22 by peripheral blood mononuclear cells from psoriatic arthritis and psoriasis patients and from healthy controls.** The concentrations (Conc) of interleukin 17 (IL-17) **(A)** and IL-22 **(B)** in culture supernatants of peripheral blood mononuclear cells from patients with psoriasis (Ps) and patients with psoriatic arthritis (PsA) and from healthy controls four days after stimulation with anti-CD3/CD28. Correlations between percentages of IL-17+ and IL-22+CD4+ T cells and the concentrations of IL-17 **(C)** and IL-22 **(D)** in culture supernatants. * p<0.05, **p<0.01, ***p<0.001.

The concentrations of IL-17 in supernatants secreted by stimulated PBMCs of PsA patients (1,287.74 ± 424.80 pg/ml) and Ps patients (1,513.12 ± 354.91 pg/ml) were significantly higher than those from healthy controls (538.60 ± 199.10 pg/ml; *P* < 0.05 and *P* < 0.05, respectively) (Figure 2A). Concentrations of IL-22 secreted by PBMCs of patients with PsA (234.20 ± 62.86 pg/ml), but not those of patients with Ps (107.70 ± 33.96 pg/ml), were significantly increased compared to healthy controls (62.86 ± 19.90 pg/ml; *P* < 0.01 and *P* > 0.05, respectively) (Figure 2B). Moreover, combining the data from all subjects tested showed a positive correlation between the percentages of IL-17+CD4+ and IL-22+CD4+ T cells with the amounts of IL-17 and IL-22 in culture supernatants (*r* = 0.5929, *P* < 0.001; *r* = 0.4324, *P* < 0.01), respectively (Figures 2C and 2D). However, the amount of IL-22 secreted by PBMCs was significantly higher in PsA patients than in patients with only Ps (256.1 pg/ml vs. 120.8 pg/ml; *P* < 0.05), even though the frequency of IL-22+ cells in PBMCs did not differ significantly in the two conditions.

### Coexpression of cytokines by CD4+ T cells in PBMCs of PsA and Ps and in healthy controls

**Figure 3:**
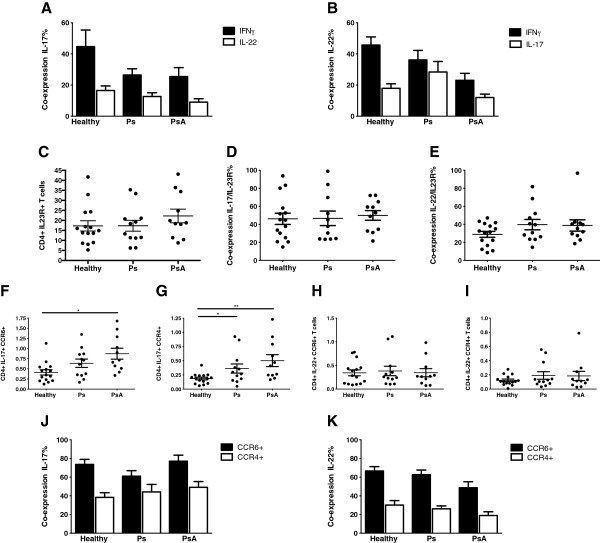
**Coexpression of cytokines and chemokines by CD4+ T cells in peripheral blood mononuclear cells of psoriatic arthritis patients, psoriasis patients and healthy controls.** Coexpression of interleukin 22 (IL-22) and interferon γ (IFNγ) by IL-17+CD4+ T cells **(A)**. Coexpression of IL-17 and IFNγ by IL-22+CD4+ T cells **(B)**. Expression of IL-23 receptor (IL-23R) by CD4+ T cells **(C)** and the proportions of CD4+IL-17+ T cells **(D)** and CD4+IL-22+ T cells **(E)** coexpressing IL-23R. Expression of chemokine receptor 6 (CCR6) and CCR4 by IL-17+ T cells **(F)** and **(G)** and by IL-22+ T cells **(H)** and **(I)** in psoriasis (Ps) patients and psoriatic arthritis (PsA) patients compared with healthy controls. Coexpression of CCR6 and CCR4 by CD4+IL-17+ T cells **(J)** and CD4+IL-22+ T cells **(K)**. * p<0.05, **p<0.01, ***p<0.001.

Cells were analysed for coexpression of the cytokines IL-17, IL-22 and IFNγ. This analysis showed that a proportion of IL-17+CD4+ T cells also produce IL-22 and IFNγ in the Ps and PsA patients and in healthy donors (IL-17+IFNγ+: 25.5% for PsA, 26.5% for Ps and 44.7% for healthy controls; IL-17+IL-22+: 9.1% for PsA, 12.7% for Ps and 16.6% for healthy controls) (Figure 3A). The same is true for IL-22+CD4+ T cells with a proportion also producing IL-17 and IFNγ (IL-22+IFNγ+: 23.1% for PsA, 36.1% for Ps and 45.7% for healthy controls; IL-22+IL-17+: 12% for PsA, 28.4% for Ps and 17.9% for healthy controls) (Figure 3B). Thus the proportions of cells making IL-17 without concomitant IFNγ or IL-22 and of cells making IL-22 without IFNγ or IL-17 (Th22 cells) was higher in both PsA and Ps patients compared to healthy donors.

### Higher proportion of CD4+IL-17+ and CD4+IL-22+ T cells coexpressed IL-23R in PBMCs of psoriasis and psoriatic arthritis patients

Very little difference in the proportion of CD4+ T cells expressing IL-23R was observed in PBMCs from patients with PsA or Ps or from healthy controls (22.20% vs. 17.34% vs. 17.26%, respectively; *P* > 0.05) (Figure 3C). In all subjects, however, a much higher proportion of the CD4+ cells making IL-17 or IL-22 expressed IL-23R (IL-17+ cells: PsA 52.9%, Ps 46.6% and healthy controls 46.1%; IL-22+ cells: PsA 38.8%, Ps 39.6% and healthy controls 31.5%) (Figures 3D and 3E).

### Chemokine expression by IL-17+ and IL-22+ T cells

Because human Th17 cells have been reported to express CCR6 and CCR4, we investigated their expression by IL-17+ and IL-22+ cells. PsA patients had increased frequency of both IL-17+CCR6+ and IL-17+CCR4+ T cells in their PBMCs compared to healthy controls (0.87% vs. 0.41%, *P* < 0.01; 0.50% vs. 0.19%, *P* < 0.05). Although patients with Ps showed significantly increased frequency of IL-17+CCR4+ cells compared to healthy controls (0.36% vs. 0.19%; *P* < 0.05), their increase in IL-17+CCR6+ cells was not significant (0.64% vs. 0.41%; *P* > 0.05) (Figures 3F and 3G). Circulating numbers of CD4+IL-22+CCR6+ and IL-22+CCR4+ were equivalent amongst the patients with Ps or PsA and healthy controls (Figures 3H and 3I). The proportion of CD4+IL-17+ T cells coexpressing CCR6 was 70.6% in all participants combined and 43.9% for CCR4. For CD4+IL-22+, the proportions were lower, with CCR6 expression at 59.3% and CCR4 at 25.1% (Figures 3J and 3K).

### Increased frequency of IL-17+ and IL-22+CD4+ T cells in psoriatic skin lesions

**Figure 4:**
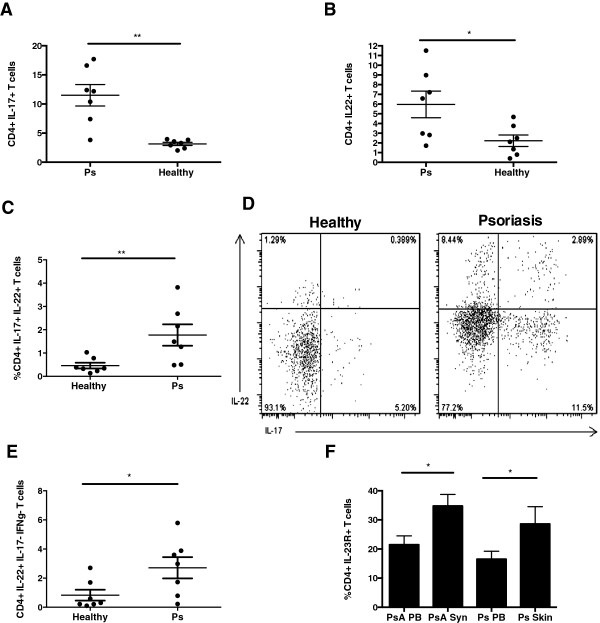
**Frequency of IL-17+ and IL-22 + CD4+ in skin resident T cells of psoriatic skin lesions compared to healthy skin and interleukin 23 receptor coexpression in skin, synovial fluid and peripheral blood of psoriasis and psoriatic arthritis patients.** Frequency of interleukin 17-positive (IL-17+) and IL-22+CD4+ in psoriatic skin lesions compared to healthy skin **(A)** and **(B)**. Frequency of IL-17+IL-22+CD4+ T cells in psoriatic skin lesions compared to healthy skin **(C)**. Representative fluorescence-activated cell sorting plots demonstrating CD4+ T-cell expression of IL-22 and IL-17 in psoriatic versus healthy skin **(D)**. Percentages of Th22 cells in psoriatic and healthy skin **(E)**. Percentage of CD4+ T cells coexpressing IL-23 receptor (IL-23R) in paired synovial fluid (Syn) and peripheral blood (PB) from psoriatic arthritis (PsA) patients and in skin and PB of psoriasis (Ps) patients **(F)**. * p<0.05, **p<0.01, ***p<0.001.

Increased expression of IL-17 and IL-22 mRNA has been reported in psoriatic skin lesions [6,22]. Psoriatic and normal skin were therefore investigated to identify resident skin T cells capable of making these cytokines. Elevated frequency of IL-17+ and IL-22+CD4+ T cells were seen in Ps compared to healthy skin (11.5% vs. 3.1%, *P* < 0.05, and 5.9% vs. 2.3%, *P* < 0.05, respectively) (Figures 4A and 4B). Coexpression of the cytokines was also examined. In Ps skin compared to normal skin, not only were the percentages of IL-17+ and IL-22+ T cells increased, but there also was a much higher proportion of IL-17+ cells which were also positive for IL-22 (Figures 4C and 4D). In addition, Th22 cells (IL-22-producing cells which do not also produce IL-17 or IFNγ) were markedly elevated in Ps skin compared to healthy skin (2.98% vs. 0.83%; *P* < 0.01) (Figure 4E). A higher frequency of T cells in psoriatic skin expressed IL-23R as compared to cells in PB of the same patients (Ps skin 28.6% vs. Ps PB 17.3%; *P* < 0.05) (Figure 4F).

Increased frequency of CD4+IL-17+ cells, but decreased frequency of CD4+IL-22+ T cells, in psoriatic arthritis synovial fluid compared to peripheral blood.

**Figure 5:**
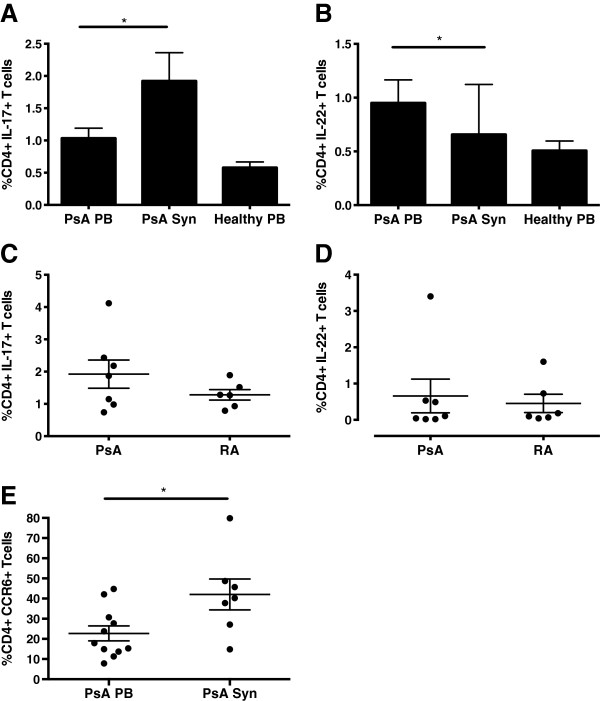
**Frequency of CD4+IL-17+ T cells and CD4+IL-22+ T cells and coexpression of chemokine receptor 6 in synovial fluid and peripheral blood of psoriatic arthritis and rheumatoid arthritis patients.** Frequency of CD4+IL-17+ T cells and CD4+IL-22+ T cells in psoriatic arthritis (PsA) synovial fluid (SF) and peripheral blood (PB) **(A)** and **(B)** and of CD4+IL-17+ and CD4+IL-22+ T cells in PsA and rheumatoid arthritis (RA) SF **(C)** and **(D)**. Percentages of CD4+IL-17+ T-cell-expressing chemokine receptor 6 (CCR6) in PB and SF of patients with PsA **(E)**. * p<0.05, **p<0.01, ***p<0.001.

No previous studies have characterized both Th17 and Th22 cells in psoriatic joints. We found higher percentages of CD4+IL-17+ T cells in PsA SFMCs compared with the same patients’ PBMCs (1.92% vs. 1.04%; *P* < 0.05) (Figure 5A), but, in contrast, the frequency of CD4+IL-22+ T cells was lower in SFMCs compared to PBMCs (0.66% vs. 0.95%; *P* < 0.05) (Figure 5B). No differences in percentages of CD4+IL-17+ and IL-22+ T cells were seen within PsA SFMCs as compared to RA SFMCs (1.9% vs. 1.3%, *P* > 0.05, and 0.66% vs. 0.45%, *P* > 0.05) (Figures 5C and 5D). Expression of both CCR6 (Figure 5E) and IL-23R (Figure 4F) was increased in the SF compared to the PB of patients with PsA.

IL-22 expression is absent in PsA ST and more highly expressed in RA ST.

**Figure 6:**
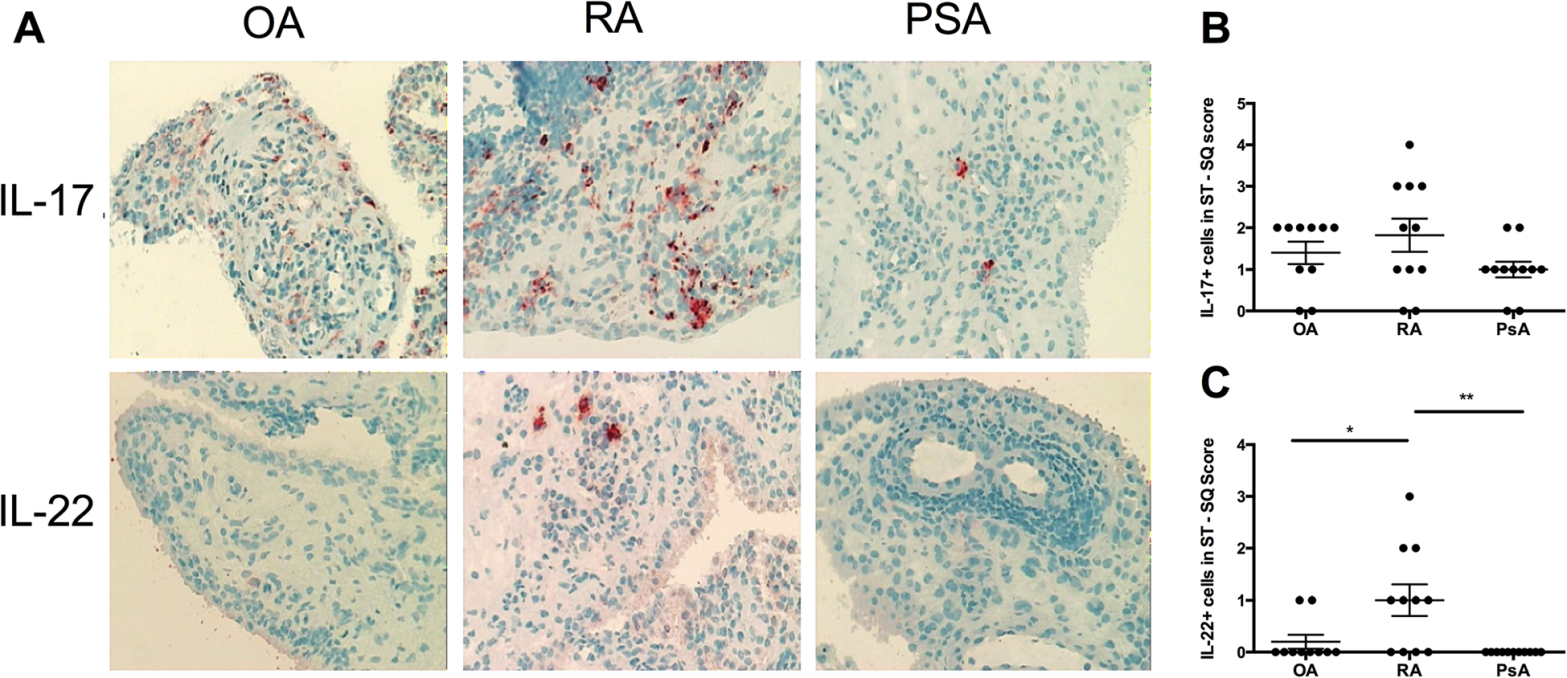
**Representative interleukin 17 and interleukin 22 expression patterns in synovial tissue from osteoarthritis, rheumatoid arthritis and psoriatic arthritis patients. (A)** Positive cells show red staining; original magnification, ×200. **(B)** and **(C)** Semiquantitative (SQ) analysis shows grading of the percentage of cells present in entire tissue: 0 = no staining, 1 = <10%, 2 = 10% to 25%, 3 = 25% to 50% and 4 = >50% for both **(B)** interleukin 17-positive (IL-17+) and **(C)** IL-22+ in tissue samples from patients with osteoarthritis (OA), rheumatoid arthritis (RA) and psoriatic arthritis (PsA). * p<0.05, **p<0.01, ***p<0.001.

Immunohistochemical staining of ST demonstrated weak positive staining for IL-17 in nine of eleven patients with PsA and in nine of eleven RA patient ST samples. By semiquantitative analysis, we found a trend toward higher expression of IL-17 in RA than in PsA, but this difference was not statistically significant (Figures 6A and 6B). In contrast, IL-22 expression was not expressed in any of 11 PsA ST samples, but was expressed in seven of eleven of the RA ST samples. By semiquantitative scoring, we found that IL-22 staining was significantly increased in RA compared to PsA and OA (Figures 6A and 6C). IL-23 was not significantly expressed in ST of patients with PsA, RA or OA (data not shown).

## Discussion

T helper (Th) cells have a central role in effecting and modulating human immune responses. Naïve CD4+ T cells commit to various subsets of Th or regulatory T cells according to the local cytokine environment and the effects of antigen-presenting cells, particularly dendritic cells, with which they interact. One subset, termed Th17 cells, was first shown to develop from naïve precursors under the influence of transforming growth factor β (TGF-β) and IL-6 ± IL-1 and to depend on IL-23 for expansion and survival. More recent work has shown that human Th17 cells can be generated by IL-6+IL-23+IL-1β in the absence of TGF-β [23].

Th17 cells produce cytokines other than IL-17 and were originally thought to be the main source of IL-22. More recent data, however, show that some CD4+ T cells make IL-22 alone, without IL-17; these cells have subsequently been termed *Th22 cells* and are also influenced by IL-23 [10,11]. IL-23, as well as its effects on the production of other cytokines, is of particular significance in relation to both Ps and PsA because genomewide association studies have demonstrated that Ps is associated with polymorphisms in genes encoding both IL-23 subunits (p40 and p19) and the *IL23R* gene; the latter is also implicated in PsA and other forms of spondyloarthropathy [24,25]. IL-23 p40 and p19 are overexpressed in Ps skin, and it has been shown that a hypofunctional variant of IL-23R is protective in psoriasis [26]. In addition to these genetic associations, there are now substantial data implicating the Th17–Th22–IL-23 axis in mouse models of both Ps and SpA [7,16,25,27-31].

IL-17- and IL-22-producing T cells have been demonstrated at various tissue sites in both PsA and Ps [8,9,32-34], but the relative contribution of Th17 and Th22 cells to each disease remains unclear. Therefore, in our present study, we sought to enumerate cells making IL-22 and/or IL-17 in skin, joint fluid and PB of Ps and PsA patients and in healthy controls and to examine their expression of IL-23R and chemokine receptors CCR4 and CCR6, which influence trafficking of these cells into skin and joints. We also pursued the expression of both IL-17 and IL-22 in synovial tissue.

We demonstrated significantly increased percentages of IL-17+CD4+ T cells in the PB of PsA and Ps patients compared to healthy controls, together with increased percentages of IL-22+CD4+ T cells in PsA patients. In Ps, the percentage of IL-22+ cells was increased, but not significantly. Likewise, increased concentrations of IL-17 were seen in supernatants of stimulated PBMCs in both PsA and Ps, whereas increased IL-22 was seen significantly only in PsA. There were positive correlations between IL-17 and IL-22 production and the frequency of IL-17+CD4+ and IL-22+CD4+ cells identified by intracellular staining, suggesting that CD4+ T cells are the principal source of IL-17 and IL-22 in the PB of both groups. These results are in general agreement with the findings of previous studies of IL-17 in PsA and Ps [8,9], but our failure to demonstrate an increased percentage of IL-22+ cells or production of IL-22 by Ps PBMCs was unexpected and has not been previously reported. One possibility is that IL-22-producing cells may be depleted from PB by recruitment to skin lesions, although in our study there was no significant difference in skin involvement as judged by Psoriasis Area and Severity Index score in Ps patients compared with PsA patients. Nevertheless, full matching of patients for extent and activity of psoriasis may not have been achieved.

In line with the hypothesis that increased production of or responsiveness to IL-23 drives increased numbers of IL-17- and IL-22-producing cells in Ps and PsA, a higher proportion of IL-17+CD4+ and IL-22+CD4+ cells expressed IL-23R. There were also significant correlations between numbers of IL-23R+ positive cells and numbers of CD4+ T cells secreting IL-17 or IL-22 (data not shown). Th17 and Th22 cells have previously been reported to express CCR6 and CCR4 [33,35], and, as expected, patients with PsA had increased proportions of both IL-17+CCR6+ and IL-17+CCR4+ cells in PB. Again, a difference was seen in Ps, where we observed increased proportions of IL-17+ CCR4+ T cells, but not IL-17+CCR6+ T cells. The explanation for this difference is unclear, but it might relate to differential recruitment to skin and joints. Although the CCR6 ligand CCL20 has been shown to be elevated and to recruit Th17 cells into inflamed joints [36,37], migration into the skin is influenced by additional factors, including ligands for cutaneous lymphocyte-associated antigen and integrins, neither of which was examined in this study.

Examination of cells in skin and SF also produced evidence of expansion of IL-23-driven T-cell subsets. In Ps skin, there were increased percentages of IL-17+ and IL-22+ cells compared to normal skin. Normal skin contained many fewer CD4+ T cells, so the numbers of IL-17+ and IL-22+ cells were very substantially increased, and, even in comparison with Ps PB, the percentages of both IL-17+ and IL-22+ cells were increased approximately tenfold. Importantly, cells producing IL-17 and IL-22 were shown to comprise three subsets: a minority producing both cytokines and distinct populations producing one but not the other. This finding emphasises the multiplicity of functional subsets with the skin. It also was reflected in PB and confirms and extends previously published data [38,39]. The proportion of IL-23R+CD4+ cells in the skin was also increased compared to PB.

In a mouse model of Ps, plaques can be induced by local IL-23 injection, and this is IL-22-dependent [28]. Therefore, the increased proportion of cells making IL-22 alone (that is, without IL-17 or IFNγ) in Ps skin compared to joints was of particular interest, given that this has not previously been studied in patients with both skin and joint disease [10]. This preponderance of Th22 cells in skin was in marked contrast to the findings in SF, where they were not elevated. IL-22-producing cells, though increased in PsA PB and expressing CCR6, might not be recruited to the joint, perhaps because of a dominant effect of skin-specific homing receptors. It has previously been reported that T cells in PsA SF lack expression of CLA, which is seen on skin T cells in Ps [40].

Further to the SF findings, immunohistochemical staining of ST from PsA and RA patients revealed that IL-22 expression was absent in all PsA samples but present in more than 60% of the RA samples. IL-22 expression has previously been demonstrated in the synovium of RA patients [41] and has been linked to the upregulation of receptor activator of nuclear factor κB ligand expression in RA synovial fibroblasts and the induction of osteoclastogenesis [42]. IL-22 expression has not been studied in PsA; however, recent work in a mouse model of SpA indicates that IL-22 may act through Stat-3, mediating osteoblastic bone remodelling, specifically at the entheseal site. Because this IL-22 expression was found even in the absence of synovitis in the mouse model [43], and since we observed a striking and specific lack of IL-22 staining in PsA ST, it will be of interest in future studies to determine whether IL-22 is also expressed in entheseal biopsies from patients with PsA.

Conversely, IL-17+ cells were clearly elevated in SF from PsA patients, and this has been noted in other forms of spondyloarthritis and in RA [9,29,30]. Weak and equivalent expression of IL-17 was demonstrated in both PsA and RA patients. This is in keeping with the findings of previous studies demonstrating that IL-17 expression is not restricted to RA but is also observed in PsA [44,45].

## Conclusion

Overall, the results of our present study strengthen the case for the central involvement of the Th17–Th22–IL-23 axis in both Ps and PsA. Th17 and Th22 cells may have common as well as divergent roles in the pathogenesis of skin and joint disease in patients with Ps and PsA, which has implications for potential treatment strategies aimed at targeting Th17 and Th22 cells or their generation.

## Abbreviations

CCR4: Chemokine receptor 4; CCR6: Chemokine receptor 6; CLA: Cutaneous leukocyte-associated antigen; ELISA: IFNγ, Interferon γ; IL-17: Interleukin 17; IL-22: Interleukin 22; IL-23R: Interleukin 23 receptor; OA: Osteoarthritis; PB: Peripheral blood; PBMC: Peripheral blood mononuclear cell; Ps: Psoriasis; PsA: Psoriatic arthritis; RA: Rheumatoid arthritis; SF: Synovial fluid; SFMC: Synovial fluid mononuclear cell; SpA: Spondyloarthropathy; SQA: Semiquantitative scoring analysis; ST: Synovial tissue; Stat-3: Signal transducer and activator of transcription 3; Th17: T helper 17; Th22: T helper 22.

## Competing interests

The authors declare that they have no competing interests.

## Authors’ contributions

HB generated and analysed the data and wrote the manuscript. PN provided clinical and technical expertise and edited the manuscript. JCG and RT analysed the data and edited the manuscript. MW, AS and OF generated and analysed the data. MDS generated and analysed the data and edited the manuscript. JSHG conceived and designed the study, analysed the data and edited the manuscript. All authors read and approved the manuscript for publication.
